# Spatial Ecology of the Critically Endangered Fijian Crested Iguana, *Brachylophus vitiensis,* in an Extremely Dense Population: Implications for Conservation

**DOI:** 10.1371/journal.pone.0073127

**Published:** 2013-09-03

**Authors:** Suzanne F. Morrison, Pita Biciloa, Peter S. Harlow, J. Scott Keogh

**Affiliations:** 1 Division of Evolution, Ecology & Genetics, Research School of Biology, The Australian National University, Canberra, ACT, Australia; 2 National Trust of Fiji Islands, Government Buildings, Suva, Fiji; 3 Taronga Conservation Society Australia, Mosman, NSW, Australia; CNRS, Université de Bourgogne, France

## Abstract

The Critically Endangered Fijian crested iguana, *Brachylophus vitiensis*, occurs at extreme density at only one location, with estimates of >10,000 iguanas living on the 70 hectare island of Yadua Taba in Fiji. We conducted a mark and recapture study over two wet seasons, investigating the spatial ecology and intraspecific interactions of the strictly arboreal Fijian crested iguana. This species exhibits moderate male-biased sexual size dimorphism, which has been linked in other lizard species to territoriality, aggression and larger male home ranges. We found that male Fijian crested iguanas exhibit high injury levels, indicative of frequent aggressive interactions. We did not find support for larger home range size in adult males relative to adult females, however male and female residents were larger than roaming individuals. Males with established home ranges also had larger femoral pores relative to body size than roaming males. Home range areas were small in comparison to those of other iguana species, and we speculate that the extreme population density impacts considerably on the spatial ecology of this population. There was extensive home range overlap within and between sexes. Intersexual overlap was greater than intrasexual overlap for both sexes, and continuing male-female pairings were observed among residents. Our results suggest that the extreme population density necessitates extensive home range overlap even though the underlying predictors of territoriality, such as male biased sexual size dimorphism and high aggression levels, remain. Our findings should be factored in to conservation management efforts for this species, particularly in captive breeding and translocation programs.

## Introduction

Most lizards that display male-biased sexual size dimorphism (SSD) exhibit some degree of territoriality or home range defense [1]. With an increasing level of SSD the effort put into home range defense by males also may increase, as territory ownership and increased body size can strongly influence access to females [2]. Female home range size and defense in such sexually dimorphic species is generally less than that of males and is influenced more strongly by access to resources [1], [2], [3]. Most territorial lizards adhere to these generalities; however, recent studies have suggested that, unlike the majority of lizards, which are insectivorous, large herbivorous lizards may play by different rules with regard to both home range size [4] and defense [5], [6].

**Figure 1:**
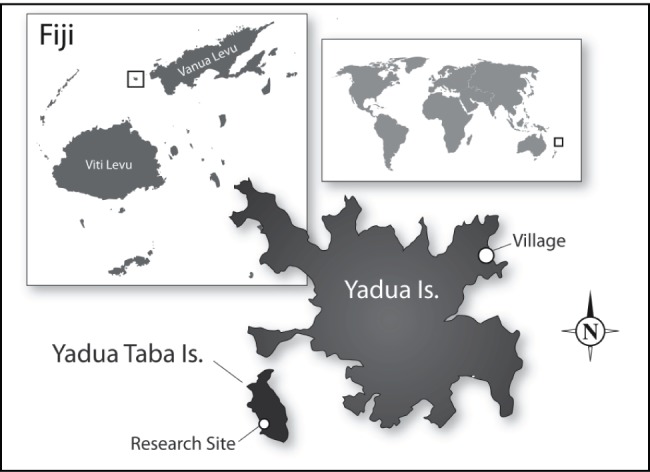
Location of Yadua and Yadua Taba Islands, Fiji, showing the village of Denimanu on Yadua and the research site on Yadua Taba.

True iguanas (iguanines) are primarily a New World group with the exception of the three living species in the genus *Brachylophus*. *Brachylophus* occur in the South Pacific Ocean archipelagos of Fiji and Tonga and are the sister clade to the rest of the iguaninae [7]. Most members of the iguaninae are large, terrestrial, and herbivorous, but the surviving *Brachylophus* species instead are medium sized (182–235 mm maximum snout vent length) and arboreal, with *B. fasciatus* (Lau banded iguana) occupying dry forests in Tonga and eastern Fiji, *B. bulabula* (Fijian banded iguana) occupying mesic forests in central Fiji, and *B. vitiensis* (Fijian crested iguana) occurring in dry forests in western Fiji [7], [8], [9]. All three species are of serious conservation concern but little is known of either *B. fasciatus* or *B. bulabula*. In contrast, the critically endangered *B. vitiensis* has been the subject of several ecological studies because it still exists in extremely high density on one small island [8], [10], [11], [12]. The nationally protected Fijian island of Yadua Taba is the last stronghold of *B. vitiensis,* and over 10,000 individuals still survive on this 70 ha island (Fig. 1) [8], [9], [10,]. This site provided an excellent opportunity to study the spatial ecology and behavioral interactions of the critically endangered iguana in its natural habitat. For herbivorous lizards living in optimal habitats, population densities can increase greatly and potentially impact on home range size [4], [13]. The impact of the extreme population density at our study site on the behavioral ecology of the Fijian crested iguana was of particular interest.

We had two major aims. First, we quantified adult male and female home ranges in an intensively studied plot within the reserve to measure the size and stability of home ranges, and investigate correlates of home range and body size in this extremely dense population. Second, we quantified injury rates of all captured iguanas and collected data on all observed behavioral interactions between adults, in order to test if residents had higher injury frequency. A detailed understanding of natural home range requirements is of particular importance if new habitats are to be identified or created for critically endangered animals.

## Materials and Methods

### Ethics Statement

All work carried out in this paper was done with approval of The Australian National University Animal Ethics Committee (approval number F.BTZ.86.05) and with permits issued by the National Trust of Fiji Islands.

### Study Site

This study was conducted on the iguana sanctuary island of Yadua Taba, in the north west Fijian archipelago (16°50′S, 178°20′E, Fig. 1). The 70 ha island is volcanic in origin, with varied terrain supporting a range of vegetation types dominated by tropical dry forest (TDF) [9], [11], [12], [14], [15]. The climate is highly seasonal with the island receiving the majority of its annual rainfall during the wet season, from November to April, though the timing and intensity of the wet season varies between years. Our primary study site was an area of TDF on the western (lee) side of the island with high densities of iguanas and iguana food plants. Minimal under-storey vegetation at this site provided clear canopy views, facilitating iguana sighting. In this area we established a 50×50 m plot, situated 50 m behind the beachfront and sheltered from it by littoral vegetation. We measured the exact location of 591 trees with a trunk diameter >5 cm at chest height. Each tree was identified with a numbered metal tag and assigned an X Y coordinate within the plot, and the position of iguanas was recorded in relation to the nearest tree.

### Mark and Recapture

Iguana mark and recapture sampling was conducted over two wet seasons during the months of October to November 2005 (9 and 11 nights each month respectively), February to April 2006 (7, 16 and 10 nights each month), and November 2006 to February 2007 (3, 15, 15 and 7 nights each month), constituting ∼455 hrs of survey time. The injury data from a subsequent dry season survey, August to September 2007 (21 and 10 nights each month) also are reported here. A minimum of two people conducted surveys at night when iguanas were sleeping, beginning soon after sunset and lasting ∼2.5 hrs. For all captured animals we recorded sex, snout-vent length (SVL) and total length (TL) to the nearest 5 mm, body mass (BM) to the nearest g, and presence and type of scars and injuries such as bite marks and missing toes, tail tips and dorsal crest spines. For males we also measured the diameter in mm of the largest femoral pore and number of pores. Iguanas were permanently marked at first capture by injecting a 12×2 mm passive integrated transponder (PIT) subcutaneously into the left flank, and we used a blue waterproof marker pen to write a unique number on the ventral surface and both flanks for subsequent visual identification. This temporary marking would last until the iguana shed its skin, and ensured that subsequent resightings could be made with no disturbance to the animals.

Approximately half of the plot area was surveyed each evening and we varied the starting location each night. Sleeping iguanas were found by spotlighting and, if novel, were captured for measurement and marking, or recorded as a resighting if visibly marked. Iguanas were caught using a telescoping 6 m fiberglass pole or by hand, and the iguana’s height above ground was visually estimated to the nearest metre. Only one position was recorded for each iguana on any night, and captured iguanas were returned to the same tree after measurement and marking. Knowing the exact position of each numbered tree on the plot meant that our positional data accuracy was very high, usually within ±1 to 2 m. In addition to recording the location of all individual iguanas, we also recorded the location and identification of all observed pairings, which we defined as an adult male and female sleeping <2 m apart.

### Home Range Calculation and Data Analysis

To estimate home range area we utilized the minimum convex polygon (MCP) method, as it is most widely reported, describes single centre ranges well, and is relatively insensitive to sample sizes [4], [16]. We initially calculated the number of sightings needed to define a home range using the 90% isopleths of the MCP with a harmonic mean peel centre [16] for adult males and females separately. The necessary number of sightings was indicated by the plot reaching a plateau at or above 80% of the final home range area [17]. Visual examination of the data indicated animals were generally using a conserved activity area with only a few outlying observations. As MCP areas are sensitive to outliers, and occasional observer error could account for some sightings outside the core area possibly exaggerating home range size, we chose to use an 85% peeled MCP. This may result in under-estimating the total habitat available to each animal but it is an appropriate measure of the overlap of animal ranges [16]. All home range analyses were conducted using the RANGES 6 program [16] and figures were generated in Arc View GIS Version 9.2.

Overlap measures were calculated in four ways for each individual [18], [19]: i) the number of overlapping adult males and females (≥190 mm SVL); ii) percentage overlap; iii) overlap pressure; and ix) encroachment. Percentage overlap is commonly used to provide a measure of the amount of a focal animal’s range shared by other individuals. Overlap pressure was calculated by combining all areas overlapping the focal home range and dividing this by the focal area. The resulting score was between 0 and n, where n = the number of overlapping individuals, and highlights areas shared by many animals. Encroachment score (0–1) describes the amount a focal individual’s range covers the ranges of other animals. Where appropriate, residuals were examined to check for normality and log transformations applied to the data if necessary. Means (x) and standard errors (s.e.) are reported throughout unless otherwise stated.

### Sexual Size Dimorphism

A sub-set of captured iguanas were measured for snout-vent length (SVL), total length, mass and three head dimensions. Head length was measured with vernier calipers (to 0.1 mm) from the posterior end of the lower jaw to the tip of the snout, and head width at the mouth axis. Head depth was measured at the centre of the head directly above the parietal scale. All morphological variables were natural log transformed before statistical analysis to meet the assumptions of normality and homogeneity of variances. Unparied *t*-tests were used to compare means of adult snout-vent length, head length, head width, head depth and mass among males and females. Analyses of covariance (ANCOVA) were then used to further investigate morphological differences between the sexes, with SVL as the covariate and sex as the nominal variable.

### Behavioral Interactions

The majority of our survey work was undertaken at night while the iguanas were sleeping. Daytime iguana interactions were observed opportunistically, at which time we recorded the nature, duration and outcome of both male-male and male-female interactions. Female-female interactions were never observed.

## Results

### Morphology and Sexual Size Dimorphism

Although male and female crested iguanas superficially appear similar in color, pattern and size [20], closer examination reveals significant sexual dimorphism. Adult males (n = 118; x = 211 mm ±0.87) reached slightly, though significantly, longer SVLs than adult females (n = 97; x = 207 mm ±0.89) (SVL: n = 215, t = 3.22, P<0.001). Additionally, at the same SVL this species exhibits male-biased sexual size dimorphism in head measurements (5–7% greater in males) and length of the longest crest spine (19–34% greater in males) (Table 1). Adult male SVL was positively correlated with BM, TL and femoral pore size (Pearson’s, n = 118; BM, r = 0.63, P<0.001; TL, r = 0.21, P = 0.02; Pores, r = 0.20, P = 0.03). Adult female SVL was positively correlated with BM and TL (Pearson’s, n = 97; BM, r = 0.65, P<0.001; TL, r = 0.38, P<0.001). Females lack functional femoral pores.

**Table 1:** Analyses of sexual dimorphism in adult *B. vitiensis.*

	Males	Females	Unpaired t test			ANCOVA							
Trait (mm)	(n = 43)	(n = 45)											
	Mean ± SE (range)	Mean ± SE (range)				Slopes				Intercepts			
			t	P		F		P		F		P	
**SVL**	206±2.4 (167–232)	204±2.3 (163–229)	−0.577	0.565									
**Jaw length**	40.3±0.45 (32.6–44.7)	38.5±0.41 (31.1–42.6)	−2.896	0.0048		0.416		0.521		42.111		<0.0001	
**Head width**	24.4±0.30 (20.4–28.6)	22.9±0.27 (17.6–26.4)	−3.621	0.0005		0.143		0.707		31.509		<0.0001	
**Head depth**	22.9±0.30 (17.8–25.2)	21.6±0.23 (17.7–24.0)	−3.335	0.0013		2.616		0.109		36.374		<0.0001	
**Max neck**	6.5±0.19 (3.8–10.3)	5.4±0.15 (3.2–7.7)	−4.392	<0.0001		2.545		0.114		29.066		<0.0001	
**spine length**													
								

Data collected from animals >160 mm SVL on Yadua Taba. Results are shown for unpaired t tests and for ANCOVA where sex is the factor and SVL the covariate. Raw data shown but all statistical tests were performed on natural log transformed data.

### Correlates of Home Range Size

During the three survey periods we captured, measured, marked and released 338 iguanas (118 adult males, 97 adult females, 123 juveniles) on our study site. For these 338 iguanas we recorded 1942 location fixes of which 1526 were for adults. Individual adults were located on 1–32 nights (X = 7±1.2). We observed 73% of adults (88 males, 69 females) in both wet seasons and 27% (30 male, 28 female) in only a single wet season. Only five of the 123 juvenile individuals were located on more than six occasions, so juveniles were not included in the analysis.

The home range measures are presented here in only two dimensions, however, it should be noted that *B. vitiensis* is arboreal and utilizes a three-dimensional habitat space. Adult iguanas have been observed at every height between the ground and the upper canopy. The mean sleeping height for marked males was 5±1.6 m (range, 0–10 m) and the mean height for females was 5.2±1.5 m (range, 0–9 m). While this adds a level of complexity to interpretation, the lack of undergrowth, small average tree size and low canopy level at the study site restricted the majority of iguana movement to a thin layer of canopy (∼3–5 m thick).

Of the 118 adult males captured in the study site, we calculated home ranges for 36 that had ten or more sightings. For females however, we calculated the home range for individuals with six or more sightings (n = 32) as only 16 individuals were recorded on more than ten occasions. We categorized adults with four or less sightings (37 males, 49 females) as non-residents and the rest as residents. We are confident about our categorization because 78% (n = 67; 29 male, 38 female) of those classified as non-residents were observed in only one wet season, while of the 68 residents, only four (6%) were seen in just a single wet season. The mean SVL and BM of male (n = 118, SVL, t = −2.58, P = 0.01; BM; t = −2.59, P = 0.01) and female (n = 97, SVL, t = −3.25, P<0.01; BM, t = −3.13, P<0.01) residents were significantly greater than for non-residents of the same sex, although at the same SVL there was no significant difference in condition (based on BM) between non-residents and residents in either sex (ANCOVA, male, F_1,73_ = 1.53, P = 0.22; female, F_1, 67_ = 2.49, P = 0.12). Male residents also had larger femoral pores than non-residents (n = 118, pores size; t = −3.44, P<0.01) and at the same SVL the difference remained significant (ANCOVA, F_1,73_ = 7.74, P<0.01). For residents only, neither SVL nor BM were significantly correlated with home range area for males (Pearson’s, n = 36; SVL, r = - 0.1, P = 0.55; BM, r_ = _0.08, P = 0.66) or females (Pearson’s, n = 32; SVL, r_ = _0.36, P = 0.46, BM; r_ = _0.23, P = 0.21).

### Home Range Size and Overlap

**Figure 2:**
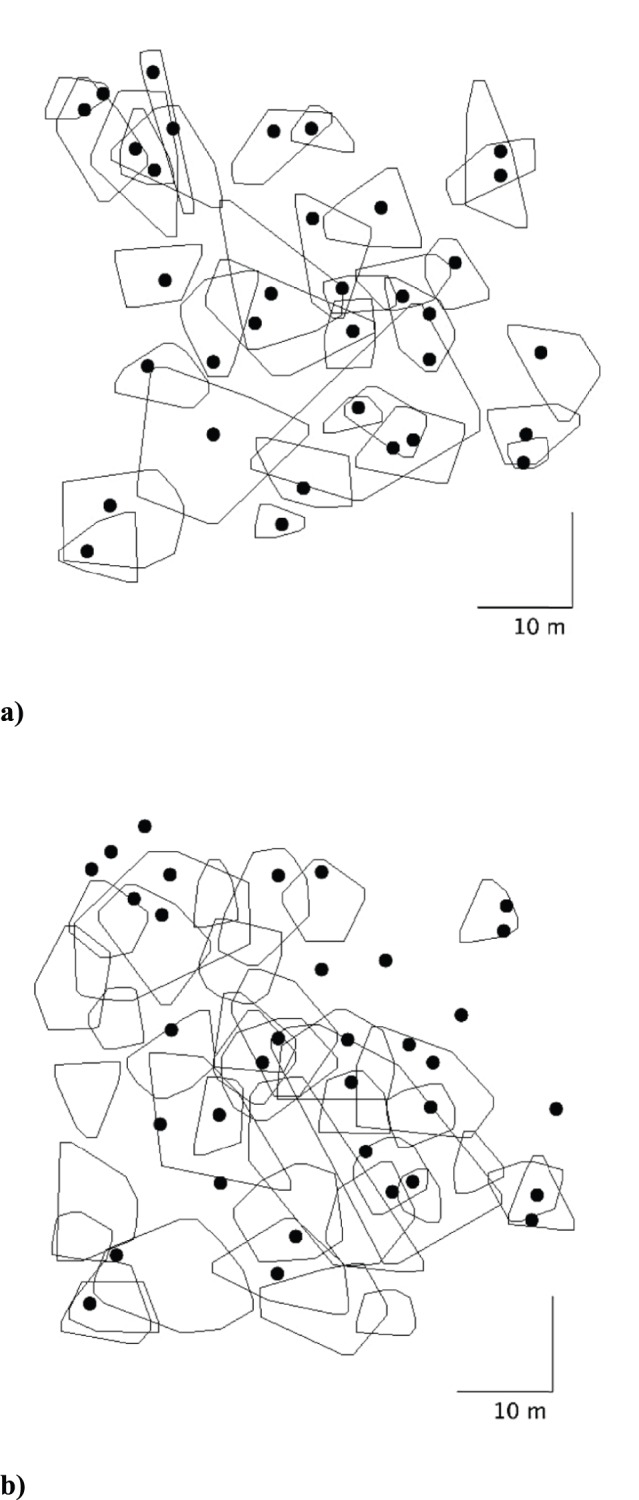
The minimum convex polygon 85% peeled core home ranges for a) adult females with ≥6 sightings (n = 32), and b) adult males with ≥10 sightings (n = 36), occupying the 50×50 m study site. The black points represent female range centers in both cases.

**Figure 3:**
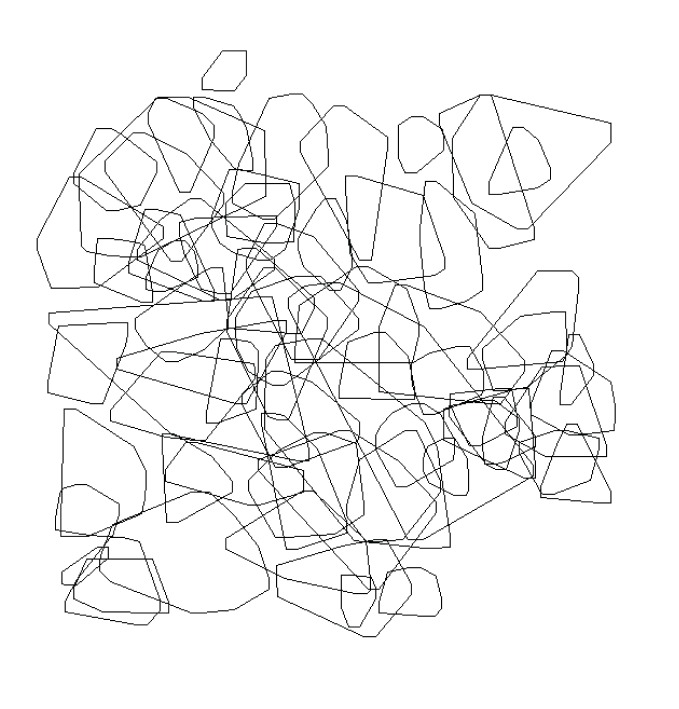
The minimum convex polygon 85% peeled ranges for all adult males with ≥6 sightings.

The mean home range size of the 36 adult male residents for which we had ten or more sightings was 64.36±8.31 m^2^, and for 32 adult females with six or more sightings was 59.87±12.63 m^2^. There was no significant difference in mean adult home range size between adult male and female residents (n = 68, t = 0.03, P = 0.76). There was extensive home range overlap within and between the sexes, with both females and males overlapping with multiple individuals (Table 2; Fig. 2). While males with ≥10 sightings were used for analysis (Fig. 2b) we also mapped the potential home range for males with ≥6 sightings to better visualize the degree of the density and overlap of animals in this population (Fig. 3). As the home range size of adult males increased, they overlapped a greater proportion of the home ranges of neighboring resident males (Pearson’s, n = 36, r = 0.62, P<0.001) and resident females (Pearson’s, n = 36, r = 0.41, P = 0.02). Likewise, female encroachment onto the home ranges of other resident females (Pearson’s, n = 32, r = 0.40, P = 0.02) and males (Pearson’s, n = 32, r = 0.38, P = 0.04) increased with focal female home range area. Heavier resident females (BM) were overlapped by fewer other females (Pearson’s, n = 32, r = −0.56, P<0.01), and males (Pearson’s, n = 32, r = −0.48, P = 0.01) than lighter females. Also, there was a trend for the percentage of a resident female’s home range covered by other resident females to decrease as BM of the focal female increased (Pearson’s; n = 32, r = −0.37, P = 0.05).

**Table 2:** Summary of home range measures in *B. vitiensis* for adult males with 10 or more sightings (n = 36) and adult females with 6 or more sightings (n = 32).

	n	Number overlapped	Percentage overlapped	Overlap pressure	Encroachment
		Mean ± s.e.	Mean ± s.e.	Mean ± s.e.	Mean ± s.e.
**F on M**	36	3.95±0.41	27.49±2.31	1.22±0.12	0.28±0.03
**M on M**	36	4.42±0.51	24.75±2.01	1.14±0.11	0.22±0.02
**M on F**	32	4.40±0.59	31.48±2.82	1.55±0.15	0.31±0.03
**F on F**	32	2.82±0.35	26.78±1.98	0.88±0.09	0.25±0.03

As an example, on the first line, a mean of 3.95 females are found in a male’s home range, 27.49% of male space is shared with at least one female, the index of overlap pressure of females on males is 1.22 and males overlap a mean of 28% of the home range of overlapping females.

### Behavioral Interactions

Combined data from three years of a broader study revealed that male and female pairs were observed in each month of the year. Maximum numbers of pair sightings were recorded in the 2006/2007 wet season (November to April). In December 2006 46.2% of the total number of adult iguanas sighted were in pairs, and in January 2007 55.1% of sighted animals were in pairs.

Of the 215 adult iguanas in the study site, 87 (40.5%) were observed in a pair at some time. Overall, 39.9% of males (n = 47) and 41.2% of females (n = 40) were observed in a pair. Males were observed paired with a mean of 1.51±0.03 different females (range 1–4) and females were observed paired with a mean of 1.78±0.04 different males (range 1–6) across the survey.

Resightings of the same individual pairs were spread across a mean period of 64.9±7.2 nights (pair combinations: n = 94; range, 1 to 557 night period) with the number of resightings of the same pair within this period averaging 2.63±0.7 sightings (n = 94; range, 1 to 12). The majority of these 94 pairs were seen together on only one occasion (n = 58, 61.7%). Of the remaining 36 pair combinations for which we had multiple sightings, 17 pair combinations (18% of all pairs) were observed across the two wet seasons.

For males, 78.7% (n = 37) of paired individuals also were residents. Looking specifically at resident males, 69% (n = 25) were observed in a pair at some time. With the exception of a single individual, all female residents were sighted in a pair (n = 31, 96%). Only 22.5% (n = 9) of females seen in pairs were non-residents. Males who were seen in a pair had significantly larger SVL, BM and pore size than non-paired males (n = 118, SVL, t = - 3.50, P<0.01; BM, t = - 2.89, P<0.01; pore, t = - 2.78, P<0.01). Similarly, females paired with a male were larger than non-paired females, exhibiting greater SVL, BM and TL (n = 97, SVL, t = - 3.87, P<0.001; BM, t = - 4.06, P<0.01; TL, t = - 1.92, P = 0.05). For male residents there was a trend for larger and heavier animals to be in a pair (n = 34, SVL, t = 1.984, P = 0.05; BM, t = 2.04, P = 0.05), and the home ranges of these paired males had fewer overlapping neighbors (n = 34, t = −2.65, P = 0.012).

We observed five instances of successful or attempted iguana copulation, 11^th^ and 18^th^ of Dec 2006 and the 16^th^, 17^th^ and 23^rd^ of Jan 2007. In two instances (17^th^ and 23^rd^ of Jan 2007) the male engaged in a protracted chase of the female through the canopy and across the ground, lasting between 20 and 30 minutes, before securing her by biting and holding the skin at her nape. Another observed copulation occurred between a pair in the canopy with no chase involved. In each case the length of copulation was greater than 10 minutes although we did not witness the entire mating sequence.

Overall injury rates, encompassing bite-marks and missing digits, crests and tail tips, were significantly higher in adults (≥190 mm SVL) than in sub-adults and juveniles (<190 mm SVL) (χ^2^ = 172.2, df = 2, n = 403, P<0.05). Sub-adult iguanas (170–189 mm SVL) exhibited significantly more injuries than juveniles (<170 mm SVL) (χ^2^ = 57.6, df = 2, n = 188, P<0.05). Male and female injury levels did not differ significantly within sub-adults (170–189 mm SVL) (χ^2^ = 1.6, df = 1, n = 57, P = 0.21), while adult males carried significantly more injuries than adult females (χ^2^ = 98.5, df = 1, n = 215, P<0.05). Evidence of recent injuries in males were greater in the wet season (82% of 152 captured individual males in Nov – April had recent injuries) than the dry season (69% of 88 males in May – Sept had recent injuries), whereas females were similar in both seasons (39% of 110 females with injuries in the wet; 35% of 63 females with injuries in the dry season). The most common injury for both sexes in all age groups were bite marks, while the biggest disparity between the sexes was in the higher frequency of missing digits seen in males (Table 3).

**Table 3:** Injury rates by sex and size class for *B. vitiensis.*

Group	n	Injury (%)	Digit (%)	Bite-mark (%)	Crest (%)	Tail tip (%)
**Adult male**	118	96	59	68	36	31
		−81.9	−50	−57.5	−30.3	−26.3
**Adult female**	97	40	13	17	11	11
		−41.2	−13.3	−17.7	−11.5	−11.1
**Sub-adult male**	34	19	7	14	4	4
		−56	−20.5	−41.1	−11.8	−11.8
**Sub-adult female**	23	9	2	7	1	0
		−39.1	−8.6	−30.4	−4.3	0
**All Juveniles (both sexes)**	131	9	2	10	0	2
		−7	−1.5	−7.6	0	−1.5

For example the first line, a total of 118 adult males were surveyed of which 96 (81.9%) had some type of visible injury, 59 (50%) had missing toes, 68 (57.5%) exhibited bite marks, 36 (30.3%) had lost at least one dorsal crest spine, and 31 (26.3%) had lost a portion of tail.

Adult female SVL and BM were significantly greater for individuals with injuries than for uninjured females. The amount of overlap pressure for males (n = 32, t = −2.25, P = 0.03) and females (n = 32, t = −2.04, P = 0.04) also was greater for injured females, and there was a trend for females with bite marks to have higher encroachment onto male home ranges (n = 32, Bite; t = −1.89, P = 0.07). A total of 82.4% (n = 34) of male residents exhibited an injury, while an injury rate of 50% was recorded in female residents (n = 32). Among male residents there was a trend for individuals with missing digits to overlap more females (n = 34, t = −1.72, P = 0.08). Among residents, injury rates were not correlated with home range area for males (Pearson’s, n = 36, r_ = _0.2, P = 0.26), though the number of missing digits was positively correlated with home range area for females (Pearson’s, n = 32, r_ = _0.55, P<0.01).

## Discussion

This study has yielded important data on the spatial organization and behavioral interactions of this critically endangered and iconic lizard. Though extirpated or declining over most of its former range [10], the population of FCI on Yadua Taba Island remains remarkably dense [9]. This density is likely to influence the frequency and form of intra and inter-sexual interactions and consequently the spatial organization and perhaps even the type of mating system. We discuss the relevance of our findings within four broad areas; correlates of resident status, home range size and overlap, behavioral interactions, and conservation implications.

### Correlates of Resident Status

As is the case in many other lizard species, FCI residents of both sexes were the largest individuals in the population. Larger male body size in lizards is advantageous in male-male competition [21], [22], presumably resulting in greater mating success and paternity [23], [24], [25]. Likewise, larger heads and dorsal spines confer advantages in combat and display, respectively [22], [26]. In tropical American green iguana *Iguana iguana*, male dominance rank, and therefore mating success, is positively correlated with head size, hormone levels and display frequency [22]. FCI exhibit the long life and indeterminate growth typical of iguanines, and with the low predation pressure on Yadua Taba [8], [12], large adult FCI are likely to maintain dominance for a number of years once this status has been reached. Based on incidental observations, Gibbons and Watkins [27] speculated that some male FCI on Yadua Taba occupied the same areas across years. We believe this to be the case for the dominant (resident) males in the population, while subordinate (non-resident) males may range more widely.

Fijian crested iguanas exhibit male-biased SSD, with males possessing larger SVLs, heads and crest spines than females. In many species of true iguana males are longer and heavier than females [2], [28]. While we do not know the specific mating system used by this species, male-biased SSD often is linked with aggression and polygynous mating systems where male mating success is skewed towards large, dominant animals [29]. We speculate that this is the case in the Fijian crested iguana but emphasize that this remains to be tested specifically. Approximately one third of adult male iguanas in this study were categorized as non-residents. These males may be ‘floaters’– an alternative mating strategy where males forego territorial defense, forage widely, and undoubtedly attempt opportunistic matings with unguarded females [19], [24], [30]. In some species subordinate male iguanas may not develop dominant male features and so remain visually similar to females. This strategy appears to reduce attack and injury and these subordinate males can sometimes enter dominant male territories without challenge [22], [24], [30]. In many cases though, subordinate status precludes males from mating opportunities, utilizing optimal foraging or basking areas, and diminishes growth and health [31], [32]. This is especially true in dense populations where dominant males are common and interactions with subordinates frequent. While the extreme density of the FCI population on Yadua Taba is likely to have some negative implications for the growth and survival of subordinate males, when SVL was held constant in our analyses we found no difference in BM between dominants and subordinates.

Male residents in the study population were significantly larger overall than non-resident males, and at equal SVL dominant males also had significantly larger femoral pores. Males of some lizard species convey a great deal of information through chemical cues, including presence of conspecifics, individual morphological traits and even health status [30], [33]; [34]; [35]. Exudates from iguanine femoral pores are most likely used in territorial marking and advertisement of male dominance [33]. In *Iguana iguana*, exudates have been demonstrated to change in composition and increase during the breeding season, most likely under the influence of androgens [33]. The diameter of *Iguana iguana* femoral pores has been positively linked to home range quality [22]. As femoral pore size also is linked to home range size in resident male FCI, this may suggest a difference in androgen/aggression levels between dominant and subordinate males. While measures of androgens and habitat quality were outside the scope of this study, we believe that femoral pore diameter may be linked to testosterone levels and social hierarchy in male FCI.

### Home Range Size and Overlap

Within the group of large dominant FCI classed as residents we found no correlates between male body size and home-range size. Within insectivorous iguanids, home range size often is linked to food availability, suggesting that larger animals have greater energetic requirements and need larger home ranges [4], [36]. However, among the herbivorous iguaninae, home range size does not appear as closely linked to body size [36]. A number of iguanid studies have demonstrated that larger males may have smaller, though more exclusive, territories than subordinate males [22], [23], and the absence of food based territorial defense in males seems to be the common pattern [36].

Unusually, we found no significant intersexual difference in home-range size between residents. Not only did we find a low male-female home range ratio but the absolute size of animal home ranges in this population was remarkably small compared to other iguanines [33], [37], [38], [39], and only slightly larger than iguanine leks (*Amblyrhynchus cristatus,* 24). Many insectivorous lizard species exhibit resource-based polygyny, where males defend an area on the basis of the food, shelter or other resources utilized by females [3]; [33]. Resource distribution obviously changes the opportunity and mode of resource defense, and it seems that the increased variability in food resources linked to herbivory has opened the way for an increased range of social systems in iguanines. Plant food resources often are less predictable spatially and temporally, than the arthropod prey of most lizards [3], [36]. Additionally, plant resources such as those on Yadua Taba can appear abundant and evenly distributed, however the nutritional quality may be low [12], [26]. Both these scenarios can render resource defense infeasible. As a result, female iguanines often are distributed in order to maximize their access to resources, and males are distributed in relation to females [32], [36], [38]. As with other herbivores in harsh habitats, FCI also are likely to be energetically constrained by the digestion of plant material and the high variability of available food over time [11], [12]. This may then impact on the size of home range a male can energetically afford to defend.

In the high-density population on Yadua Taba, encounters between FCI are common. While overlap levels in this population were high and suggest frequent interactions, they did not reach the >50% territory overlap level reported for iguanines with passive social structures or dominance hierarchies [37]. Often where space is limited, or encounters between animals within a population are frequent, males will abandon defense and form relationships based on a social hierarchy [13]. In this population however, overlap levels fell within the spectrum of home-range defense commonly seen in iguanine species [37]. While males were aggressive towards other males, neither sex appeared to exclusively defend their home range. Stamps [3] summarized that for lizards, dominant male herbivores are not as aggressive as insectivores, and generally allow subordinate males within their territories or maintain exclusive territories of only a small area. In the desert iguana, *Dipsosaurus dorsalis*, high density and increased aggression saw the increased use of exclusive core areas inside larger, extensively overlapping, home ranges [13]. Even within iguanine species where dominant males exclusively defend areas from other adult males, female-impersonating subordinate males may gain access to dominant territories [3], [23], [24], [32], [40], [41].

FCI exhibit relatively slight male-biased SSD and the sexes appear superficially similar, to the point where it was originally believed that they differed significantly only in the larger size of the male femoral pores [20]. We observed subordinate FCI males remaining green, as would a female, when facing a dominant male’s display; these males may avoid excessive aggression and injury in this manner. In this way high levels of overlap may occur between, though not generally within, male age and size cohorts. Most male iguanines are territorial, displaying overt aggression towards rival adult males, and it was no surprise that male residents in this population overlapped more with females than with other males. Similarly, females overlapped more with males than with other females. Although female FCI home-ranges were overlapped by a number of different males, this may not have translated into more association with these males. Female Cuban iguanas, *Cyclura nubila*, were vigorously defended by dominant males even though other males overlapped the area [32]. As with males, female spacing also could be facilitated through intrasexual aggression. However, this was not observed in the field, and in other iguanine species female-female aggression is minimal and generally limited to nest-site defense [3]. It has been suggested that female iguanines are generally non-aggressive when there is no defendable limiting resource [28].

In the absence of aggression, one possible mechanism maintaining the relatively low female-female overlap could be their avoidance of harassment by subordinate males. In this scenario dominant males defend females within their territories from subordinate ‘floater’ males seeking opportunistic matings. Other lizard studies indicate females with male ‘minders’ may enjoy uninterrupted foraging and maintain condition better than harassed females [24], [42], [43]. In the dense population on Yadua Taba males may be limited in the number of females or the area they can defend, and females may be spaced out to maximize protection and limit harassment. It also is likely that with such a dense population, female home ranges are approaching, or have reached, the smallest area required for continued fitness. However, without details on habitat quality and levels of male harassment, we can only speculate that FCI follow the general pattern where females are spaced to maximize fitness and male spatial organization is based on maximizing mating opportunities with females [44]. Further study is needed to elucidate whether males in this population defend resources utilized by females, females themselves, or both.

### Behavioral Interactions

Over the course of this study just under half of all adult iguanas were observed in an intersexual pairing on at least one occasion. Of these animals the vast majority were also residents, possessing the attributes of larger size, mass and, for males, femoral pore size. Among male residents there was a trend for larger and heavier males to pair with females, and for these males to have fewer overlapping neighbors. Though female choice in reptiles remains relatively uncommon [45], female preference for larger males has been noted in a number of lizard species [24], [42], [46]. In the green iguana *Iguana iguana,* large dominant males perform 90% of copulations, with associations between males and females lasting several weeks. Female green iguanas copulate multiple times during the season but generally only with one male [23]. In FCI, both males and females were observed in a pairing with more than one other individual, suggesting multiple mating for both sexes.

Both wild and captive male FCI are aggressive to male conspecifics [27], [47], and injury rates for adult male FCI were high in this population, with 80% of males displaying at least one type of injury. In the first natural history notes on the species, Gibbons observed that male FCI on Yadua Taba were aggressive during the breeding season and that serious injuries could be inflicted during aggressive disputes [27]. His observation of around 40% of males suffering toe loss is similar to the 50% found during this study, and, likewise, Gibbons noted that females and juveniles exhibited few such injuries. We found no relationship between adult male injury levels and any other home range or morphometric measure. Because FCI are long-lived, males may obtain permanent injuries when young which then remain into dominancy. Aggressive interactions occur more frequently in higher density populations [13], [36], [38], and it is likely that the high injury rates recorded in this population are an artifact of high density. However, as the FCI on Yadua Taba represent the only extant sustainable population, and the only one ever studied, we have no basis for comparison between injury rates in populations of different densities.

### Conservation Implications

Our study indicates that male FCI are highly aggressive when in dense populations. In addition to the general statement that adult male FCI should be separated in captivity to reduce injuries and stress, we suggest that juvenile males nearing maturity also should be housed separately from adults. It has been demonstrated in a number of lizard studies that dominant males can suppress the development, reduce growth rates and decrease survivorship of young and subordinates males [31]. In the wild population, males >170 mm SVL exhibited an increasing injury rate indicative of agonistic encounters, most likely with older males. As Yadua Taba remains the site of the only secure FCI population in Fiji - and the world - there is an urgent need for the establishment of additional populations to safeguard this species’ survival [48]. Translocation of FCI from Yadua Taba to other suitable islands remains a priority. Our findings on FCI intraspecific interactions provide information that may assist in maximizing male genetic contribution to any FCI translocated populations, and minimizing stress and injury to any captive and transported animals. Ongoing surveys of translocated populations also have the potential to greatly assist in answering questions relating to the dynamics of populations at differing densities.
